# Team behaviour in interprofessional collaboration during trauma alerts: A critical incident study from the perspective of radiographers

**DOI:** 10.1111/scs.13308

**Published:** 2024-11-06

**Authors:** Marice Bäckström, Katarina Leijon‐Sundqvist, Lise‐Lott Lundvall, Karin Jonsson, Åsa Engström

**Affiliations:** ^1^ Division of Nursing and Medical Technology, Department of Health, Education and Technology Luleå University of Technology Luleå Sweden; ^2^ Department of Nursing Umeå University Umeå Sweden

**Keywords:** collaborative practice, core competence, shared decision‐making, teamwork, trauma care, trauma team

## Abstract

**Introduction:**

Challenges in mutual awareness in interprofessional collaboration (IPC) along with relational and cultural barriers among professionals disrupt flow and delay decision‐making in trauma care. Thus, this study explores team behaviours within IPC in trauma teams during trauma alerts from the perspective of radiographers.

**Methods:**

A qualitative approach was used with a critical incident technique (CIT) design applying interviews with radiographers within three hospitals in Sweden from May 2022 to May 2023. CIT analysis was conducted with an abductive approach, applying an IPC core competency framework.

**Results:**

The results present collaborative requirements in trauma care from radiographers' perspective narrating a distribution of team behaviours within trauma team collaboration and fundamental skills in IPC. Behaviours within interprofessional values and ethics were the most reported incidents related to valuing radiographers' contributions to IPC in acute trauma care.

**Conclusion:**

Exploring behaviour through critical incidents associated with core competencies of IPC highlights the importance of interprofessional values as a foundation for successful IPC in the trauma team. The results show deficiencies in inclusive behaviour, influenced by the hierarchical environment of IPC. Power imbalances in this setting are traced to differences in perceived value and shared understanding among team members, possibly rooted in professional identity and culture. A dedicated leader is argued, as the recognition of radiographers' scope of practice in trauma imaging, emphasising the significance of shared decision‐making.

**Clinical Implications:**

The findings highlight organisational and relational coordination challenges for optimising competencies in IPC. IPC's success requires reinforcing values and ethics by empowering members' contributions and shared decision‐making. This involves clarifying and recognising responsibilities, particularly for radiographers, ensuring their role in trauma imaging is respected and integrated into decision‐making.

## INTRODUCTION

This paper explores radiographers' perspectives on interprofessional collaboration (IPC) in trauma teams. IPC is an approach in which various professionals collaborate to provide high‐quality care across settings, coordinating specific knowledge and responsibilities in an integrated process of interdependence [1]. Both teamwork and IPC call for a movement from individualisation to collective competencies [2, 3], where the interdependence of the team is associated not only with the necessary individual professional competence but with the collective competence of the combined knowledge and team capabilities needed for successful team performance [4]. Multiple models of IPC competence show similar content, albeit with variations in definitions of competencies and skills [5]. The framework for IPC developed by McLaney et al. [6] proposes a collective approach, with collective competencies including associated team behaviours that foster and support collaborative practice. The core competencies allocated in this framework include *interprofessional (IP) values and ethics*, *IP conflict resolution*, *reflections*, *communication*, *role clarification*, and *shared decision‐making*, along with behaviours providing specific actions that collectively promote a collaborative practice.

The IP trauma team combines the specialised competencies of professionals from the emergency, anaesthesia, intensive care, surgery and radiology departments responding to an ad hoc team activation, resulting in a variety of team staffing in critical situations under time constraints [7]. Trauma care and trauma radiology have been highlighted as a complex, high‐pressure environment that demands round‐the‐clock availability [8]. In this setting, radiographers ensure rapid and safe diagnostic that guides decision‐making related to injuries, measures and treatments, managing high volumes of imaging under critical circumstances while maintaining high‐quality care in a patient‐safe manner [9]. However, as IPC depends on an integrated process of interdependence, an ad hoc team structure hinders the development of team identity and mutual trust, where team performance depends on coordination, team dynamics and team behaviours [10]. Furthermore, interpersonal relationships, tasks and responsibilities and team processes are seen as related to IPC conflicts [11]. The presence of relational and cultural barriers among professionals disrupts flow and delays decision‐making and crucial interventions in trauma care [12]. Whereas previous research highlights the importance of radiographers' interaction in trauma teams for effective IPC and successful team performance, a lack of shared knowledge in trauma teams impedes patient safety and hinders timely and accurate decision‐making [13]. Exploring team behaviours and IP competencies deepens our understanding of the behavioural patterns and competencies that promote or hinder an inclusive and effective working environment in trauma teams. Therefore, this study aimed to explore radiographers' perspectives on team behaviours within IPC in trauma teams during trauma alerts.

## METHODS

### Design

The study adopted a qualitative design using the critical incident technique (CIT) by Flanagan [14] and an abductive approach [15]. CIT includes observing and analysing human behaviours and their consequences in a specific situation to understand how incidents influence performance and outcomes [14]. In this study, a critical incident (CI) was defined as a behaviour that affected team performance and the outcome of IPC. The study is reported following Tong et al.'s [16] consolidated criteria for reporting qualitative research (COREQ).

### Participants and setting

A purposive selection was used to collect experiences from radiographers participating in trauma team activation (TTA) and responding to trauma alerts. Participants were recruited from hospitals with known accessibility to emergency care and trauma radiology. Registered radiographers were requested in three hospitals, in different parts of Sweden, with frequent trauma alerts and various trauma organisations. In one of the hospitals, the trauma radiology department and emergency department (ED) were located on the same floor, while in the other two hospitals, the departments were separately located.

### Procedure

A letter with information about the study and voluntary participation was sent to the heads of the radiology departments and team leaders, who forwarded the invitation to radiographers participating in TTA. A respondent‐driven sampling was subsequently used where participants suggested potential radiographers who could participate but had not responded; those potential participants were then contacted by email. Finally, 10 radiographers participated in individual interviews, whose demographic characteristics are presented in Table 1.

**TABLE 1:** Demographic characteristics of participants (*n* = 10).

Demographic	Frequency
Gender, female, *n* (%)	6 (60%)
Age, median (range)	46 (28–67 years)
Years of professional work experience, median (range)	16 (3–41 years)
Years of experience in ETI and TTA, median (range)	11 (1–31 years)

Abbreviations: ETI, emergency trauma imaging; TTA, trauma team activation.

### Data collection

Data collection occurred from May 2022 to May 2023 through interviews conducted by the first author. A pilot interview was conducted to test the interview guide. Data were collected inductively using a semi‐structured interview guide (Appendix S1) that addressed the study aim; ‘critical incidents’ were changed to ‘significant events’ to better reflect collaborative events without implying a serious incident in patient care. This adjustment aligns with recommendations in healthcare studies [17]. The pilot interview enriched the data collection by providing several CIs in IPC during trauma alerts and was included in the study. In CIT, participants are asked to describe CIs reproduced from memory, which can be enhanced and verified if information and clarification are provided before collection [14]. Before the interview, the participants received information on key definitions such as IPC and the definition of significant events to induce memories of when they had been involved in trauma alerts recalling events in IPC that affected the outcome of collaboration positively or negatively. The interviews were conducted via video (Zoom), except for one by phone, and all interviews were recorded and transcribed verbatim by the first author. Interviews ranged from 39 to 72 min (average 47 min). Data saturation in CIT is determined by the number of CIs, with a minimum of 100 incidents recommended for comprehensiveness [14].

### Data analysis

The analysis was carried out according to CIT as described by Flanagan [14] to increase the value of the data without diminishing the study's scope, specificity and validity, and the concept was further developed following Viergever [15] by conducting an abductive analysis. The first author conducted the analysis; to increase validity, each step in the analysis was discussed among the authors to reach a consensus. In the first step, the CIs were identified according to the aim by analysing each interview separately, extracting CIs inductively so as not to disregard important behaviours in this context. In the second step, an abductive approach was used where each CI was condensed and labelled in terms of *behaviours* and *consequences of the behaviour*. The labelling of behavioural codes followed the associated behaviour based on the framework of core competencies in IPC [6]. The identified critical incidents were coded and sorted according to the framework's structure, based on our interpretation of the behaviours' meaning. The framework outlines general core competencies and associated behaviours. In the analysis, the related behaviours appeared shorter and more contextually adapted to the framework. After coding, the CIs were sorted by similarity and difference in each behaviour code. Following Flanagan [14], conducting the concluding step of interpretation and determining the degree of generality of the data, the analysis provided a comprehensive understanding of the data in the context of team behaviour within IPC core competencies. The third and last step accounted for general behaviour by moving between the specificity and generality of the data's valid interpretation [14], and the abductive analysis adding an exploration and in‐depth understanding of the behaviours in context, following Viergever [15]. The results are presented in main areas through a distribution of behaviours within the core competencies of IPC [6]. The number of incidents was counted and presented in the distribution and pattern of incidents, following Flanagan [14].

### Ethical considerations

Ethical aspects and risks were considered throughout the study process. Confidential measures were implemented following the principles outlined in the declaration of Helsinki [18]. Before the interviews, written informed consent was secured from all participants and they were thoroughly briefed about the study, with an emphasis on the voluntary nature of their participation and their ability to withdraw from the study. All participants gave their informed consent before the data collection, and the first author who conducted the data collection had no relation or superior position to the participant. The study obtained ethical approval from the Swedish Ethical Review Authority [2021‐03544].

## RESULTS

Participants described a median of 30 CIs (10–70 per participant) out of a total of 349 identified incidents within IPC. Fourteen behaviours related to the 19 behaviours included in the framework [6] were identified. The frequency of CIs, including both positive and negative aspects of the incidents, sorted for behaviours under each core competence [6], is presented in Table 2. The description of the behaviours associated with the core competencies in the framework has been concretised in the table to illustrate the behaviours identified in the data and related to the framework.

**TABLE 2:** Frequency of critical incidents (*n* = 349).

Core competence	Associated behaviour	Frequency *n* (%)
**Interprofessional values and ethics**		**145 (41.5%)**
	Considers the value and ethics of team members	113 (32.4%)
	Collaborates willingly	19 (5.4%)
	Establishes a safe environment	13 (3.7%)
**Communication**		**65 (18.6%)**
	Considers members’ needs	35 (10.0%)
	Exchanges information	27 (7.7%)
	Uses common language	3 (0.9%)
**Role clarification**		**63 (18.1%)**
	Articulates their role	55 (15.8%)
	Recognises limitations	6 (1.7%)
	Seeks understanding	2 (0.6%)
**Interprofessional conflict resolution**		**43 (12.3%)**
	Listens open‐mindedly	20 (5.7%)
	Discusses and agree on solutions	12 (3.4%)
	Addresses issues	11 (3.2%)
**Shared decision‐making**		**27 (7.7%)**
	Identifies and designates accountability	27 (7.7%)
**Reflection**		**6 (1.7%)**
	Dedicates time to team reflection	6 (1.7%)

### Interprofessional values and ethics

Behaviours within IP values and ethics included the most reported incidents and were seen as a foundation of IPC from the radiographers' perspective, shaping IPC and the success of trauma care. The behaviours that exemplified these values included a cooperative attitude and a willingness to collaborate, which established a more balanced power structure in the hierarchical environment and created a safe environment in the team. For instance, when team members actively sought input from the radiographer or deferred to their expertise during discussions, they exhibited behaviours such as considering the value of the radiographer as part of the team.

Some physicians rely entirely on what you should do or what you think. The physician looked at my name tag and said – Okay, let's listen to [person's name], what should we do? It feels good to feel trusted. At the same time, it's nothing strange because we know what we're doing and how we should do it. It's bizarre that something like that should feel special. (Participant 5)

The value of the radiographer as an integral part of the trauma team was enhanced through respectful behaviour and a considerate approach towards their area of expertise. This was evident when the team acknowledged the radiographer's contributions, leading to more efficient collaboration. For example, implementing the radiographer's suggestions not only sped up the care process but also reinforced the importance of their role in the team. However, participants consistently pointed out a lack of awareness regarding the radiographers' need for concentration during decision‐making stages in the imaging and care procedures. This lack of awareness led to delays or errors, affecting the overall quality of patient care. Such behaviour, or lack thereof, manifests as interruptions or distractions that hinder the radiographer's focus and task performance. These disturbances negatively impacted the IPC's effectiveness and diminished the radiographer's perceived value. Behaviours that undermined an inclusive approach, such as disregarding input from the radiographer or not allowing them to voice their opinions, were contrary to the principles of inclusivity and respect. These behaviours prevented all team members from contributing to the decision‐making process and establishing a safe environment.

Furthermore, the hierarchical environment negatively influenced inclusive and respectful behaviour. For instance, when the team leader and other team members dominated discussions or made decisions without consulting or regarding the radiographers, it created a power imbalance and a culture that discouraged open communication and collaboration. This not only hampered the team's effectiveness but also undermined the value of each member's unique expertise and perspective. Insufficient respect for the radiographer's knowledge and responsibilities within the team resulted in nonchalant behaviour towards their tasks, which was not conducive to an open climate and was seen as inhibiting a safe environment. Participants described the absence of awareness and the lack of trust in their competence as causing inquisitorial behaviour from other team members when the radiographers suggested certain actions. The questioning, in turn, led to disputes, delaying the care and preventing the radiographers from fulfilling their tasks and responsibilities.

The physician in charge of the patient usually questions why the CT takes such a long time. They don't understand that we're doing an extended examination that takes time to plan. Often, they [radiologists] have a request in patient arm positioning when we examine the thorax and abdomen to reduce interference, and to produce the best possible examinations, resulting in a discussion of stating and explaining, after which the physicians still don't understand the importance. In case of bleeding somewhere, it is difficult to distinguish what it is from an artefact [disturbance in the image]. Unfortunately, you will be mistrusted. (Participant 1)

In summary, creating a safe environment for all team members required behaviours that encouraged transparency and an inclusive attitude where values and ethics were considered to increase trust and willingness to cooperate within the team. By actively promoting these principles, hierarchical barriers were overcome through an inclusive approach, working together towards common goals while respecting each other's competencies.

### Communication

Behaviours within IP communication, associated with considering members' needs, exchanging information and using a common language, were reported as the second most common incidents. Behaviours within IP communication contributed to a shared understanding of the situation when team members searched for and ensured awareness of the situation through the radiographer's perspective. Participants described behaviours such as proactively seeking awareness and asking for and exchanging information or advice from the radiographer as behaviours that facilitated collaboration and fostered specific and timely communication. Furthermore, behaviours of validation could be verbal or through actions, hence considered radiographers' needs, such as assisting with the setup of the equipment. This collaborative behaviour not only lightened the radiographer's workload but also ensured that the entire team was aligned regarding the patient's care. When the radiographer suggested a specific course of action, other team members communicated the radiographer's needs demonstrated their understanding and assisted in implementing this action, further enhancing the team's effectiveness.

However, participants also described preparing under unclear conditions, hence the team leader and other team members unintendingly withheld necessary information from the radiographer. Behaviours of not sharing specific and timely information about the patient and the situation were particularly prominent in cases where the radiographer was absent from pre‐arrival team briefings and initial care when the patient arrived at the ED. The use of unfamiliar terminologies, interpreted as a lack of common language, was described as occasionally occurring among new or inexperienced trauma leaders, leading to confusion and uncertainty. Additionally, not receiving vital information created frustration and hindered the planning of care for the specific patient or other patients needing prioritisation.

You call to get information on what has happened, how many people are involved, children or adults, trying to get an overview to plan. I'll ask for information and report to my colleague and then we wait for them to get back to us. Sometimes there is a long delay before they reconnect and we feel awkward recalling, − how are you doing, where are you? Sometimes the trauma alert can be cancelled, and we need to know to make other plans. It may be the second time you call, sometimes it can be too much for them. At the same time, standing by, waiting and nothing happens. We can't admit another patient until we know how the patient is doing in the ED. Suddenly, you've been waiting for half an hour and then it's announced that the trauma alert has been cancelled. Maybe you have some idea of the patient's condition, maybe you can call and say that we can examine another patient in between and they'll get back to you. (Participant 10)

Although participants described actions that occurred simultaneously, patiently waiting for the other healthcare professionals to carry out their responsibilities as a matter of procedure, allowing each team member to work undisturbed and communicate one at a time. In the initial care in the ED, the participating radiographers described behaviours of waiting for directives from the trauma leader to perform their measures and tasks. This behaviour ensured that the radiographers' actions aligned with the overall plan of care and maintained the hierarchical structure of the team. In the absence of directives, the radiographers reported having to make themselves heard and point out the need for action, such as proactive behaviours of ensuring and proposing radiographic examinations or preparatory measures for the CT examination.

You must obtain your place and make yourself heard to be present. You observe discussions of trauma imaging and planned measures, but no one engages in direct contact with you. (Participant 4)

In summary, effective IP communication was essential to ensure participation and understanding within the team. By actively seeking understanding and exchanging specific information with the radiographer, the need for involvement can be met to improve the quality of care. Behaviours that hindered safe and effective communication were seen to cause frustration and barriers in the care, emphasising the importance of clear directives, using common language and mutual awareness to maintain an effective and safe care process.

### Role clarification

Behaviours within role clarification, associated with articulating their role, recognising limitations and seeking understanding, were reported as the third most common incidents. Behaviours of role clarification were depicted as crucial for the success of IPC within trauma teams. Despite this, radiographers were describing a lack of clarity in the division of roles and responsibilities. Team members exhibited behaviours of lacking knowledge and understanding of each other's scopes and expertise, resulting in behaviours where they questioned radiographers' responsibilities, thus hampering IPC. This was evident when the team leader did not include or articulate radiographers' tasks and when radiographers described instances where others stepped into their responsibilities in patient care resulting in conflicts and argumentations. Moreover, the radiographers described having to take control of the situation by clarifying and articulating their roles. Furthermore, insecure behaviours from uncertain leaders led to unclear leadership steering the radiographer to a call for accountability from an invisible leader, needing to actively seek understanding of the roles of others portrayed as leading to time‐consuming discussions in the team. By contrast, participants described directive trauma leaders behaving as capable of coordinating collaboration in a critical situation. Such leaders were described as exhibiting behaviours such as recognising their limitations, seeking understanding and sharing the leadership by explicitly stating the radiographer's responsibility, thus facilitating both the examination process and the overall IPC. Participants perceived the assumption of a leadership role as a move that requires authority, courage and experience.

When things get out of hand, it can be due to an inexperienced leader. It's not that you end up in a dispute right away, but it prolongs the [care] time, and some steps are repeated because others have not perceived what was done or said. People were running around, which was perceived as a stressful situation, which led to more errors. This is when I'm starting to count down [loudly] for the CT to start, although it may not be my job, I have sometimes stepped in and generated an action. (Participant 2)

In summary, role clarification was critical to successful IP collaboration within trauma teams. Members who were able to articulate their role and/or scope of practice facilitated collaboration and optimised patient care. Actively seeking understanding from others helped to coordinate efforts and reduce inefficiencies. Effective leaders recognised their limitations, consulted with each other and delegated responsibilities to the radiographer, strengthening collaboration and the shared pursuit of high‐quality care. Conversely, ambiguity in these roles led to delays and inefficiencies.

### Interprofessional conflict resolution

Behaviours within IP conflict resolution associated with listening open‐mindedly, discussing and agreeing on solutions and addressing issues were reported as the fourth most common incidents. Behaviours of IP conflict resolution were associated with team values and ethics, as conflict resolution reflected the valuation of their own and other's contributions to the collaboration. Addressing important aspects of the radiographer's responsibilities and emphasising their needs for action were seen as necessary but difficult in conflict situations; however, supporting behaviours from others strengthened the courage to speak up and facilitated a discussion when it was difficult to interfere. This was particularly evident in the initial care, where the radiographer was perceived to have an observing role, searching for specific information or needing to interact and when other team members emphasised and addressed issues from the radiographer's perspective. Within the CT examination, conflicts frequently led to negotiations and time‐consuming discussions that created difficulties moving forward, where a lack of understanding and behaviours of inattentiveness to other points of view hindered the conflict resolution and in turn the IPC. Contrary, when team members listened openminded to radiographers' directives and explanations the conflict was resolved and collaboration progressed. Nevertheless, as ambiguities of procedure are managed through calm behaviour by serenely dealing with criticism and anger and agreed solutions developed, the relationship between professionals evolved.

A surgeon requested the image plates, and I explained that we have only one, a new digital detector, and he got incredibly upset and shouted about it. After the radiographic, I turned to the same doctor and calmly asked him if he wanted to see the image because it was digital now. He was amazed that it could be viewed directly and was very impressed and satisfied, which completely changed the tone and atmosphere in the room. It was like breaking down a barrier between us. It was a great experience, going from being so incredibly upset to being grateful instead. The collaboration was much better after that. He was a well‐known doctor who could be quite harsh and unresponsive. Through that, we got a better relationship in future meetings. (Participant 4)

In summary, IP conflict resolution was closely linked to team values and ethics, reflecting the values and contributions of each team member. Supportive behaviour from colleagues increased the courage to speak up and facilitated discussion. Behaviours of open‐mindedness to others' points of view, and addressing issues from the radiographer's perspective, facilitated the resolution of conflicting situations. Unresolved issues in conflicts led to negotiations and protracted discussions, which hindered progress due to a lack of understanding and inattentiveness to different points of view. However, when ambiguities were addressed, and solutions agreed professional relationships improved.

### Shared decision‐making

Behaviours within shared decision‐making associated with identifying and designating accountability were one of the least reported areas of incidents. Shared decision‐making emerged in the analysis through association with designated responsibilities and shared leadership in the IPC, also related to role clarification and collaborative discussions within conflict resolution. Shared decision‐making occurred when the radiographer articulated actions during the imaging examination and the members exhibited behaviours of mutually discussed solutions to achieve a quality examination. Otherwise, participants reported apparent behaviour of leader‐follower where the leader directed and supported the team, focusing on team effectiveness, while team members followed directives and decisions. However, the radiographers described lacking information about decisions where other team members contributed crucial information in a delayed manner or not at all, interpreted as inadequate designated accountability, somewhat excluding the radiographer from the decision‐making process. Contrarily, to some extent, a clear transfer of responsibility within CT examinations was interpreted as behaviour contributing to a form of shared leadership and shared decision‐making. In certain situations, the trauma leader delegated accountability and decision‐making related to the imaging process to the radiographer, and team members exhibited behaviours such as following the radiographer's instructions.

On these occasions, someone clearly shows that they are the trauma leader in charge. It makes a difference how well things run when someone is the clear leader, and you can easily communicate with that person. When they invite you to take the leadership role in the CT room, as opposed to when they retain leadership, the outcome of the collaboration becomes much smoother. (Participant 4)

In summary, shared decision‐making and responsibility, achieved by identifying and designating accountability within IP trauma teams, were crucial for achieving team efficiency. However, challenges remained in ensuring sufficient information and a clear distribution of responsibilities within the teams. Shared decision‐making was recognised as an essential component of IPC, involving all team members in decision‐making processes to foster participation in mutually discussed solutions and enhance team performance, ultimately ensuring the best possible care outcomes.

### Reflection

Behaviours within reflection associated with dedicating time to team reflection were one of the least reported areas of incidents. Participants briefly mentioned IP team reflection, but their narrative demonstrated a need for common reflective procedures where coordinators and team members included the radiographers in the team briefing. Improvements in inclusive behaviour and attitudes towards the radiographer were observed after mutual team exercise, including an enhanced understanding of the radiographer's responsibilities. All participants expressed a desire to dedicate time for shared meetings and team exercises, but the lack of invitation left a feeling of not being requested. Nevertheless, participants sought inclusion in the trauma team exercises due to their desire to highlight different aspects of a conflict.

Usually, the radiology department doesn't participate in the debriefing that the ED conducts. (Participant 4)

You might think that you could communicate and reflect together as a team, maybe in some way you could have discussed the special situation that arose, but we didn't do anything special. They were kind of satisfied with the examination [although an incident occurred], and then they left. (Participant 8)

In summary, the desire for shared meetings and team exercises, the commitment to ongoing reflection and the behavioural improvements observed demonstrate an understanding of team dynamics and development and a desire to optimise teamwork and team functioning in the IPC.

## DISCUSSION

This study aimed to explore team behaviours within IPC in trauma teams during trauma alerts from the perspective of radiographers. The study connects CIs with core competencies in IPC and applies a structured framework, yielding a deeper understanding of radiographers' perspectives on critical behaviours within IPC in trauma care. The findings emphasise the importance of IP values and ethics as a foundation for successful collaboration as highlighted with the most recurring core competence, with behaviours related to valuing radiographers' contributions in IPC in trauma care. Participants described a wide range of team behaviours: to some extent, they felt respected and acknowledged, but in other ways not valued for their knowledge and responsibilities in the team. Previous studies have shown that the trauma team structure includes an inner and an outer core team including an active/passive role in patient care [19]. Whereas some include radiographers in the core team [20] others observed a relegation of radiographers to the outer team as supporting staff until a radiographic examination is ordered [19]. Furthermore, radiographers are perceived as having a tentative role in trauma teams, occasionally being included in the core team depending on the course of the trauma care, while in other situations being excluded accordingly from the decision‐making [13].

This study presented critical situations of IPC when undesirable team behaviours contradicted an inclusive approach and when hierarchical structures created power inequalities that affected how radiographers were valued within the team, in turn affecting the team's attitude, values and trust. Furthermore, this power imbalance affected communication, decision‐making and the overall effectiveness of the collaboration, which in turn affected the patient safety and quality of trauma care. These findings show that the power imbalances in a hierarchical environment stem from differences in the perceived value and shared understanding among team members, thus aligning with Noyes' [21] finding that perceived power differences related to professional identity and culture influence attitudes and beliefs regarding IPC. IPC in healthcare often encounters ethical conflicts due to differing perceptions of patients' roles and the diverse understanding of each professional's responsibilities in the care process. Similar to our findings, Pakkanen et al. [22] found that undervaluing other professionals' expertise indicates a lack of respect and results in feeling disregarded and sidelined in the collaboration.

Furthermore, ethical conflicts may arise when there is a lack of commitment to collaboration among the involved professionals, resulting in disregarding shared values [22]. Our result showed a restored power balance when a team leader promotes open and unrestricted communication among all members reinforcing the value of radiographers' contributions and ensuring the utility of all expertise. However, inattentive behaviour to other points of view can be explained by an individual's strong sense of power that often overlooks the potential knowledge gaps of others, which can hinder a smooth IPC. Hence, anticipating others' needs and adapting behaviour accordingly is crucial for effective functioning [21]. When team members perceive higher conflict potential and power differences, IP communication and coordination suffer because behaviours such as speaking up, seeking help and understanding perspectives are inhibited [21]. Furthermore, both addressing and resolving conflicts depend on individuals' perceived inherent power [23]. This is in line with our findings of waiting behaviour in some conflict situations. On the contrary, our results also showed critical situations wherein team members exhibited supporting behaviour by noticing and addressing the radiographer when they sought information or called for action. The supportive engagement in IP teams [24] influenced by power and hierarchy reflects the team's trust, professional status and perceived power, which can result in apparent threats on the one hand or a culture conducive to speaking up on the other.

Our results demonstrate that IP communication played a crucial role in establishing a shared understanding. Behaviours such as proactively seeking understanding, asking for information or guidance from the radiographer and addressing communication promptly were highlighted as factors that enhanced collaboration. Additionally, the behaviours illustrating team situational awareness encompass both cognitive and behavioural processes. These processes ensure that all team members possess adequate information and exchange details about one another, facilitating team awareness. With this knowledge, the team can develop more effective work strategies, assigning tasks to those most suited for them [25].

We found that leadership was integral to both role clarification and shared decision‐making. An apparent leader‐follower relationship was perceived to impact IPC, where the leader directed and supported the team, focusing on team effectiveness, while team members followed directives and decisions. Similar to our findings, successful leadership in trauma teams includes direct communication and decisiveness, where decisions are integrated by input from team members [26]. Our results convey, to some extent, a clear transfer of responsibility within CT examinations, interpreted as a form of shared leadership and shared decision‐making. In certain situations, the trauma leader delegated accountability and decision‐making related to the imaging process to the radiographer. Along those lines, during the critical phase of trauma care, the competence of an assigned trauma leader in team management with supportive actions influences team effectiveness in a positive way [27]. On the one hand, a strong hierarchy in the trauma team with a dedicated leader is preferred; on the other hand, there is also a perceived need to be involved in decision‐making and information sharing to make important decisions within the radiographer's area of responsibility. Furthermore, the CIs reported within IP reflection were few in frequency but emphasised the need for team reflections within the temporarily assembled teams to achieve high‐quality care. By promoting IPC across organisational boundaries, research shows that IPC with coordination and knowledge sharing is needed, where consistent opportunities for interaction between team members can develop the ability to mutually share goals, share knowledge and develop mutual respect [28, 29].

### Strengths and limitations

CIT can be described as a retrospective narrative, where a limitation of the method is that CIs reflected on from memory likely relate to exceptional, often negative events [15]. The interviews explored behaviours that had consequences and effects on the team performance and outcome of IPC and resulted in both descriptions of desirable and undesirable team behaviours. The results are presented as main areas through a distribution of the behaviours within the core competencies of IPC [6]. One limitation is that the associated behaviours in the framework were adapted to the data and context of trauma care. As a result, some behaviours described in the framework were not represented in our data. The data were collected through an inductive approach with open‐ended questions, not influenced or directed by the framework. It is unclear if some non‐identified behaviour might be relevant or prominent in the specific context studied. If we had asked about core competencies and associated behaviours, we might have gathered different data. Nonetheless, the data provided a deeper understanding of positive and negative events that affected IPC, highlighting that some behaviours and core competencies were more represented than others in this context. Presenting the distribution of incidents provides insight into the frequency and pattern of crucial behaviours and barriers, which may help prioritise actions, increase transparency and improve the quality of trauma care. The research involved various trauma organisations enhancing the dependability in multiple contexts as well as the transferability to other professionals in temporarily assembled teams collaborating in complex environments. The research team, consisting of radiographers and nurses with experience in trauma alerts and teamwork, used a critical awareness of our preconceptions and experiences, with a reflexive approach to be open to alternative interpretations. To ensure the validity of the process, all authors discussed each step of the analysis to reach a consensus. However, external triangulation was not conducted, which could be considered a limitation.

## CONCLUSION

Exploring behaviour through CIs associated with core competencies of IPC highlights the importance of IP values and ethics as a foundation for successful IPC in the trauma team. The study identified various situations that are critical for radiographers' inclusion influenced by the hierarchical environment of acute trauma care. Power imbalances in this setting were traced to differences in perceived value and shared understanding among team members, possibly rooted in professional identity and culture. A clear and dedicated leader is important, as is the recognition of radiographers' scope of practice in trauma imaging, emphasising the significance of shared decision‐making and shared leadership. These elements are vital for effective collaboration and impact on patient safety and quality of trauma care.

## CLINICAL IMPLICATIONS

The findings emphasise team performance relying on mutually dependent participants and highlight challenges in organisational and relational coordination for optimising the competencies of IPC. The success of IPC requires reinforced IP values and ethics by empowering members' opportunity to contribute to collaboration and decision‐making. This involves clarifying and recognising responsibilities, particularly for radiographers, ensuring their role is respected and integrated into decision‐making. The findings show a need to highlight interprofessional competencies and appropriate behaviours in education and clinical practice. Future studies should explore other team members' perceptions of collaborating with radiographers and consider organisational perspectives for a comprehensive understanding of IPC in trauma care.

## AUTHOR CONTRIBUTIONS

**Marice Bäckström:** conceptualisation, methodology, data collection, analysis, writing the original draft. **Katarina Leijon‐Sundqvist**, **Lise‐lott Lundvall and Karin Jonsson:** conceptualisation, analysis, review and editing, supervision. **Åsa Engström:** conceptualisation, methodology, analysis, writing, review and editing, main supervision. All authors approved the final version.

## FUNDING INFORMATION

The research was conducted as part of a doctoral project at Luleå University of Technology. The university provided support through the doctoral program but was not involved in the study or manuscript writing, editing, approval or decision to publish.

## CONFLICT OF INTEREST STATEMENT

The authors declare that there is no conflict of interest.

## ETHICS STATEMENT

The study obtained ethical approval from the Swedish Ethical Review Authority, and all participants gave their informed consent prior to data collection.

## Supporting information


Appendix S1

## Data Availability

Research data are not to be shared due to the confidentiality of participants.
