# Supplementary material for: Team behaviour in interprofessional collaboration during trauma alerts: A critical incident study from the perspective of radiographers
Source: Scand J Caring Sci. 2024 Nov 6;39(1):e13308. doi: 10.1111/scs.13308 (PMC11686041; doi:10.1111/scs.13308)
Supplement: Supplementary file 1 — Appendix S1 [file SCS-39-0-s001.docx]

**Appendix S1.** Semi-structured interview guide

The interview guide contains probing questions about significant events (critical incidents) exploring behaviours that had consequences and effects on the performance and outcome of IPC. The questions are repeated when the participant has described the event in detail to obtain several described events from the same participant.

Probing questions

If you remember a trauma alert situation where you and other professionals were involved when a significant event affected the collaboration:

What was the situation that led to the collaboration (context)?

How did you act (*behaviour*) in the event?

How did the other team members act (*behaviour*) in the event?

How was the event, that affected the collaboration, handled by you and the other team members? (*behaviour*)

What was the outcome of the collaboration? (*outcome*)

How did the event affect the collaboration between the professionals in the team? (*outcome*)

What was the outcome for the patient care and patient safety? (*outcome*)

What do you think made the collaboration turn out the way it did?

What in this event makes you consider it as a significant event that affected the collaboration?

**Can you give further examples of significant events/situations:**

- Can you give an example of something more in that situation or another***event*** that **contributed positively**to the collaboration between different professions in trauma alerts? E.g., that made a successful collaboration.

- Can you give an example of something more in that situation or another***event*** that **negatively** **affected** the collaboration between different professions in trauma alerts? E.g., that made an unsuccessful collaboration.

Is there anything missing from the description that you would like to highlight or add?
